# Recent Advances in the Use of Exosomes in Sjögren’s Syndrome

**DOI:** 10.3389/fimmu.2020.01509

**Published:** 2020-08-06

**Authors:** Yupeng Huang, Ruicen Li, Sheng Ye, Sang Lin, Geng Yin, Qibing Xie

**Affiliations:** ^1^Department of Rheumatology and Immunology, West China Hospital, Sichuan University, Chengdu, China; ^2^Health Management Center, West China Hospital, Sichuan University, Chengdu, China

**Keywords:** Sjögren’s syndrome, exosomes, immune response, biomarkers, treatment

## Abstract

Sjögren’s syndrome (SS) is a chronic autoimmune disorder of the exocrine glands mediated by lymphocytic infiltrates damaging the body tissues and affecting the life quality of patients. Although traditional methods of diagnosis and treatment for SS are effective, in the time of personalized medicine, new biomarkers, and novel approaches are required for the detection and treatment of SS. Exosomes represent an emerging field in the discovery of biomarkers and the management of SS. Exosomes, a subtype of extracellular vesicles, are secreted by various cell types and can be found in most bodily fluids. Exosomes are packed with cytokines and other proteins, bioactive lipids, and nucleic acids (mRNA, circular RNA, non-coding RNA, tRNA, microRNA, genomic DNA, and ssDNA), and transport such cargo between cells. Evidence has indicated that exosomes may play roles in processes such as the modulation of the immune response and activation of inflammation. Moreover, due to features such as stability, low immunogenicity and toxicity, long half-life, and the capacity to penetrate the blood-brain barrier, exosomes have also emerged as therapeutic tools for SS. In this review, we summarize existing literature regarding the biogenesis, isolation, and function of exosomes, specifically focusing on exosomes as novel biomarkers and their potential therapeutic uses in SS.

## Introduction

Sjögren’s syndrome (SS) is a chronic autoimmune disorder of the exocrine glands. It is characterized by lymphocytic infiltration in the salivary and lacrimal glands (LGs) resulting in oral and eye dryness. Extraglandular manifestations such as musculoskeletal pain, fatigue, and systemic features also develop in a significant percentage of patients. This exocrinopathy can occur alone (primary Sjögren’s syndrome, pSS) or secondary to another autoimmune disease such as rheumatoid arthritis (RA), systemic lupus erythematosus (SLE), and systemic sclerosis (SSc). The antinuclear antibody (ANA) is the most frequently detected autoantibody in SS, while anti-Ro/SSA and anti-La/SSB are the most specific prognostic markers (1–3). The prevalence of SS is 0.29–0.77% overall and 3–4% among the elderly. The ratio of male to female cases is 1:9, and the average age of onset is over 50 years. 5% to 10% of the patients can develop non-Hodgkin’s lymphoma, the most serious complication of SS, within 10 to 15 years of follow-up (4, 5). Despite extensive research on the underlying cause of SS, the pathogenesis remains obscure. Multiple factors, including the environment and the immune system, may contribute to the development of this disease.

In the last decade, researchers have focused their attention on the release of extracellular vesicles (EVs). These double lipid bilayer-enclosed membranous vesicles are produced and discharged from almost all cells and represent more than the casual dispersal of cellular “dust” (6). Further, EVs deliver complex chemical messages over long distances (7). Exosomes, the most well-known and studied subtype of EVs, were first described as nanoscale vesicles derived from various normal and neoplastic cell lines in the 1980s (8, 9). These endosome-derived nanovesicles have a characteristic cup-shaped morphology as observed under electron microscopy, with a diameter of 30–100 nm and a density between 1.13 g/ml and 1.19 g/ml. They exist in numerous bodily fluids, including serum, saliva, urine, cerebrospinal fluid, milk, and tears, under both normal and pathological conditions (10, 11). Exosomes are packed with various components and have the capacity of inducing functional responses in recipient cells (12–15). Through the transfer of bioactive molecules between cells, exosomes mediate intercellular signaling and participate in various physiological and pathological processes (16). The involvement of exosomes in the development and treatment of autoimmune diseases has also been extensively researched (17, 18). Lee et al. demonstrated that circulating exosomes in patients with SLE could be associated with disease activity and might therefore serve as biomarkers of disease activity (19). Kimura et al. found that circulating exosomes suppressed the induction of regulatory T cells *via* let-7i-mediated blockade of the IGF1R/TGFBR1 pathway in multiple sclerosis (20). Few reviews have investigated and summarized the functions of exosomes in SS. In this review, we will focus on the recent advances regarding exosomes in SS and their potential as biomarkers and therapeutic tools.

## Exosomes

### Exosome Biogenesis and Isolation Methods

The generation of exosomes is initiated by invagination of the plasma membrane to form endocytic vesicles. When these newly formed endosomes mature, depressions in the endosomal membrane take place, and intraluminal vesicles are produced. Intraluminal vesicles are further transformed into multivesicular bodies (MVBs) with a dynamic subcellular structure, also known as late endosomes. MVBs then merge with the plasma membrane and release the vesicles contained within, called exosomes. Exosome biogenesis is complex and tightly regulated by multiple factors. The endosomal sorting complex required for transport (ESCRT) is the principal protein family governing the synthesis of exosomes. Downregulation of ESCRT-0 and ESCRT-0 proteins decrease exosome secretion. Conversely, depletion of ESCRT-I proteins increase exosome production. Moreover, exosomes can be generated without ESCRT proteins, and ESCRT-independent machinery may contribute to the sorting of cargo into exosomes. Lipids also play a crucial role in the biogenesis and transport of exosomes. Several other proteins, including GTPase proteins and lactadherin, are also involved in the biogenesis of exosomes (16, 21, 22). Nevertheless, mechanisms of exosome biogenesis and secretion require further elucidation.

Exosomes are secreted into biological fluids which contain other vesicle types such as microvesicles and apoptotic bodies. It is therefore necessary to isolate exosomes from contaminating material. The isolation of pure exosomes is essential for understanding their mechanisms of action and potential applications. Several methods have been adopted for the isolation for exosomes: differential centrifugation, ExoQuick^TM^ extraction kits, sucrose density gradient ultracentrifugation, and immunoaffinity sedimentation (21). These methods may have certain limitations, such as low yield and purity. Microfluidics-based technologies have recently become available for the isolation, detection, and analysis of exosomes and do not have the above-mentioned limitations (16).

Accurate evaluation of the physicochemical characteristics of exosomes, such as size, shape, and density, is crucial for exploring the biological interactions of these vesicles. Western blotting, enzyme-linked immunosorbent assay (ELISA), real-time quantitative polymerase chain reaction (RT-qPCR), dynamic light scattering (DLS), fluorescence-based detection, nanoparticle tracking analysis (NTA), atomic force microscopy (AFM), and transmission electron microscopy (TEM) are commonly used techniques for exosome characterization (23, 24). Western blotting and ELISA are used for the identification of intra-vesicular or membrane protein markers (25), while RT-qPCR is used for the detection of exosome-related RNA (26). NTA, AFM, and TEM have been developed to determine the size, density, morphology, and composition of exosomes (27, 28). Recently, a new technique, tunable resistive pulse sensing (TRPS), has been used to measure the size distribution and concentration of exosomes (29). To discriminate between exosomes from normal and pathological cells, considering their inherent heterogeneity, we need to combine quantification techniques. This will open up new avenues for exosome detection and characterization.

### Composition and Function of Exosomes

Exosomes have a lipid bilayer structure and are released upon fusion of the MVB with the plasma membrane (30–32). Exosomes contain various proteins (e.g., cytokines, GTPases, Alix, TSG101, tetraspanins, heat shock proteins, and integrins), lipids (e.g., phosphoglycerides, cholesterol), and nucleic acids (e.g., mRNA, circular RNA, non-coding RNA, tRNA, microRNA, genomic DNA, and ssDNA) (33–38). Due to their lipid bilayer, genetic information and other transported components are protected from degradation (31). Exosomes are secreted by various immune cells [e.g., T cells, B cells, dendritic cells (DCs), and macrophages] and non-immune cells (39). Once released, exosomes can interact with specific recipient cells based on the expression of adhesion molecules, such as phosphatidylserine receptors, integrins, and glycans on the exosome surface (40, 41). Thus, information can be transmitted to target cells *via* exosomes.

The existence of EVs had been reported as early as 1946 (42), and De Broe described the release of these “membrane fragments” as a general characteristic of viable cells in 1977 (43). In 1983, a major discovery by Harding and Johnstone revealed that transferrin receptors were associated small 50 nm-sized vesicles. Through endocytosis and recycling, these vesicles were released from maturing blood reticulocytes into the extracellular space. The term “exosome” was coined by Rose Johnstone to describe these EVs (16). In 1996, researchers found that Epstein-Barr (EB) virus-transformed B lymphocytes had the capacity of releasing exosomes, inducing major histocompatibility complex (MHC) class II-restricted T-cell responses (44). In the early days of EV research, exosomes were simply considered as cellular waste disposal units. In more recent years, however, exosomes have been intensively researched and have been shown to act as mediators of immune stimulation and modulation (45). Exosomes regulate multiple immune processes, including antigen presentation, T-cell activation and polarization, and immune suppression (46, 47). Immune cell-derived exosomes have been studied extensively. For example, MHC-I and MHC-II molecule-carrying exosomes derived from antigen-presenting cells stimulate CD8 + and CD4 + T cells, respectively (48). Further, exosomes secreted from macrophages infected with bacteria had pro-inflammatory effects on naïve macrophages, promoting the maturation of DCs (49). It should be noted that the release of exosomes by DCs and B lymphocytes is increased after cognate T cell interactions, indicating that the secretion of exosomes by immune cells could be regulated by the cellular environment (50, 51). Exosomes secreted by non-immune cells, such as mesenchymal stem cells (MSC) and tumor cells, have also gained attention. MSC-derived exosomes are capable of enhancing the differentiation of immunosuppressive cells and inhibiting the proliferation of natural killer (NK) cells and T cells (52). Recent research has reported that exosomes from bone marrow-derived mesenchymal stem cells (BMSC) regulate the polarization of macrophages in rat models (53). In addition, exosomes derived from tumor cells can inhibit the activation of T cells *via* programmed death-ligand 1 (PD-L1) (54). Among exosome-associated bioactive components, microRNAs (miRNAs) not only modulate gene expression in immune cells, but also have immunological functions (55, 56). Okoye et al. suggest that miRNA-containing exosomes secreted from primary regulatory T cells suppress Th1 cell responses (57). Ismail et al. found that macrophage-derived exosomal miR-223 induced the differentiation of recipient monocytes (58). Another study showed that miR-223 promoted the invasion of breast cancer cells *via* the Mef2c-β-catenin pathway (59). Other functions of exosomes have also been investigated, including regulation of the incorporation of neurons and glial cells in the central nervous system (60, 61) and thrombosis in the cardiovascular system (62–64). A previous review summarized the involvement of exosomes in: (1) protection against viruses and bacteria; (2) regulation of tumor immunity; (3) mediation of immune suppression by tumor cells (65). In general, the function of exosomes depends on the status of host cells and tissue. Studies have shown that exosomes play significant roles in angiogenesis, antigen presentation, apoptosis, coagulation, inflammation, and intercellular communication through the transfer of bioactive molecules such as RNA and proteins. Further, exosomes are involved in both normal and pathological processes, including cancer, infections, and autoimmune diseases.

Exosome carrying specific molecules of interest could act as potential biomarkers. Exosomal biomarkers can be divided into three groups: tumor-derived exosomes, exosomal surface proteins, and exosomal nucleic acids (66), and these indicators can provide insightful information for the early diagnosis of cancer and other diseases. For example, exosomes containing proteoglycan glypican-1 (GP1) may be potential biomarkers for pancreatic cancer (67). Exosomes loaded with CD81 have a potential role in the diagnosis of hepatitis C and the evaluation of treatment responses (68). Exosomes carrying a specific kind of phosphorylated amyloid peptides are promising biomarkers for Alzheimer’s disease (69). Some unique characteristics of exosomes have attracted the interest of researchers, including their stability under long-term storage, low immunogenicity and toxicity, their ability to protect encapsulated components, and their capacity for penetration of the blood-brain barrier (BBB) (70–73). Thus, exosomes could potentially be used as nanocarriers for various nucleic acids, proteins, and small molecular drugs (74). Some antineoplastic agents, such as doxorubicin and paclitaxel, could be encapsulated and delivered via exosomes to treat brain tumors (75, 76). Tian et al. revealed that curcumin-carrying engineered exosomes induced the suppression of the inflammatory response and cellular apoptosis in lesion regions of ischemic brains (77).

There are various studues investigating exosomes in autoimmune diseases, among which studies of rheumatoid arthritis (RA) have been the most thorough. With regard to pathogenesis, in the synovium of RA patients, synoviocyte-derived exosomes, which contain citrullinated autoantigens, may promote synovitis and cartilage damage (78, 79). In contrast, exosomes from neutrophils that have infiltrated into inflamed joints are protective factors for chondrocytes (80). From the perspective of treatment, BMSC-secreted exosomal miR-192-5p can delay inflammation in RA (81). Mesenchymal cell–derived miRNA-150-5p–containing exosomes and MSCs-derived miRNA-124a-overexpressing exosomes are also expected to be involved in potential therapeutic strategies for RA patients (82, 83). Information about the role of exosomes in the pathogenesis or their possible use for treatment of other autoimmune diseases has been scarce in comparison to RA. It has been suggested that exosomes from inflamed intestinal cells and renal tissue have pathogenic roles in ulcerative colitis and lupus nephritis, respectively (84, 85). Lu et al. showed that BMSC-derived exosomes carrying miR-223-3p attenuated autoimmune hepatitis in a mouse model (86). Neutrophil-produced exosomes from systemic sclerosis patients have the ability to inhibit the proliferation and migration of endothelial cells (87).

## Exosomes in SjÖgren’s Syndrome

### Role of Exosomes in the Pathogenesis of SS and as Potential Biomarkers

In 2005, Kapsogeorgou et al. reported that salivary gland epithelial cell (SGEC) lines from SS patients secreted significant amounts of exosomal vesicles, similar to those from non-SS subjects. These SGEC-derived exosomes contained detectable amounts of epithelial-specific cytoskeletal proteins, as well as anti-Ro/SSA, anti-La/SSB, and Sm ribonucleoproteins. Although secretion was not restricted to SS-derived cells, this was the first time that SS-specific autoantigens were detected in exosomes, indicating that exosomes may participate in the presentation of intracellular autoantigens to autoreactive lymphocytes, as part of the development of SS (88). Another study showed that a functional EB virus miRNA, ebv-miR-BART13, can be transferred from B cells to SGECs, affecting salivary secretion (89). More recently, Cortes-Troncoso et al. suggested that T cell-derived exosomes containing miR-142-3p may be a pathogenic trigger of SS. When transferred into SGECs, miR-142-3p-carrying exosomes can affect intracellular Ca^2+^ signaling and decrease cyclic adenosine monophosphate (cAMP) production, thereby leading to glandular cell dysfunction (90). At present, studies of exosomes in SS mainly concentrate on tears and saliva (Table 1), as such fluid samples can easily be obtained using a simple, non-invasive, and safe method. Because SS is a disease affecting multiple organ systems, investigation of exosomes in other tissues and organs is still required.

**TABLE 1:** A selective overview of studies reporting exosomes in Sjögren’s syndrome.

**Articles**	**Origin of exosomes**	**Exosome components**	**Role in pathology**	**As a biomarker**	**Potent therapeutic effect**
(88)	SGECs		+		
(89)	B cells	ebv-miR-BART13-3p	+		
(90)	T cells	miR-142-3p	+		
(93)	SGECs, lacrimal gland cells			+	
(94)	SGECs	miRNAs		+	
(95)	SGECs	miRNAs		+	
(96)	DCs				+
(97)	DCs				+
(98)	MSCs				+
(100)	Placenta tissue	miRNAs of C19MC			+
(87)	human umbilical cord MSCs				+

*SGECs, salivary gland epithelial cells; DCs, dendritic cells; MSCs, mesenchymal stem cells; miRNA, microRNA; C19MC, chromosome 19 microRNA cluste.*

**FIGURE 1:**
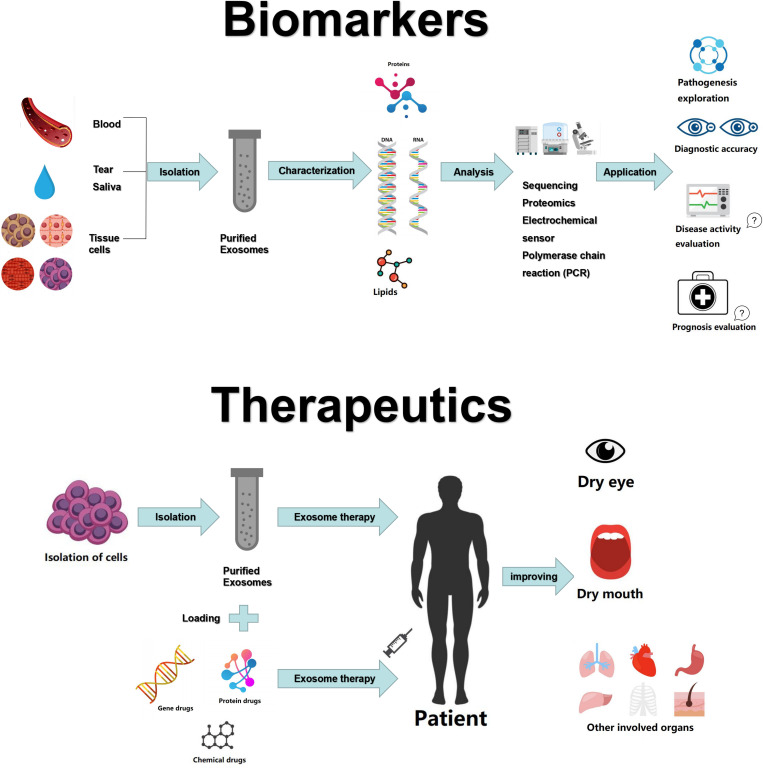
Exosomes as biomarkers and therapeutic tools in Sjögren’s syndrome.

The international consensus criteria for SS includes ocular symptoms, oral symptoms, objective evidence of dry eyes and salivary gland involvement, as well as laboratory test abnormalities (91). The presence of ANA has some merit for the detection of SS, but 31.7% of healthy individuals may also be positive for ANA (92). Rheumatoid factor (RF) is not specific to SS, as it is also upregulated in other autoimmune diseases, especially RA. Anti-Ro/SSA antibodies have good specificity and can be found in two-thirds of SS patients, often at the same time as anti-La/SSB antibodies (91). However, sometimes during the early stage of the disease, patient symptoms are not typical, and even the serological examination is not positive. Therefore, a more accurate diagnostic method is required. Aqrawi et al. isolated EVs (including exosomes and microvesicles) from saliva and tear fluids of patients with SS and utilized liquid chromatography-mass spectrometry (LC-MS) for the detection of potential biomarkers (93). Michael et al. were the first to isolate exosomal miRNAs from the parotid saliva of SS patients, proposing that the miRNA content of salivary exosomes could provide markers for the diagnosis of various salivary gland diseases, such as SS (94). Similarly, Alevizos et al. showed that salivary gland miRNA expression patterns precisely distinguished SS patients from control subjects, suggestive of the potential of miRNA for the detection of inflammation or salivary gland dysfunction in SS (95). Despite these promising findings, there is not enough evidence for the use of exosomes or exosomal miRNAs as reliable markers for SS. Future experiments may refute some of the current findings. Moreover, the use of exosomes for evaluating SS disease activity and prognosis has not yet been investigated in studies (Figure 1). Thus, further research is required to confirm the potential roles of exosomes or exosomal miRNAs as robust, specific, and sensitive biomarkers for SS.

### Exosomes as Therapeutic Tools for SS

The management of SS is long-term and complex. Saliva substitutes and artificial tears could be used to relieve symptoms. Non-steroidal anti-inflammatory drugs (NSAIDs), hydroxychloroquine, and corticosteroids are effective for the treatment of SS. Other powerful immunosuppressants, such as methotrexate, mycophenolate mofetil, and biological agents, are also required (91). However, long-term use of these drugs can cause a number of adverse effects. Fortunately, exosomes have been intensively studied for their potential use in autoimmune diseases. Kim et al. suggested that injection of exosomes secreted from DCs treated with interleukin-10 (IL-10) inhibited the onset of collagen-induced arthritis in a mouse model and reduced the severity of arthritis (96). Exosomes derived from indoleamine-expressing DCs had immunosuppressive and anti-inflammatory effects in an arthritis model (97). Bai et al. reported that exosomes from MSCs efficiently attenuated autoimmune uveitis in a murine model (98).

A study by Li et al. has demonstrated that administered exosomes derived from human umbilical cord MSCs efficiently eased ophthalmitis in a model of human SS (99). Bullerdiek et al. reported that analogs of chromosome 19 miRNA cluster (C19MC)-derived miRNAs could be applied in clinical practice for autoimmune conditions such as SS (100). Ocular involvement is one of the main manifestations of SS. The most commonly used treatment for eye disease is topical instillation of eye drops. However, there are some limitations, including quick clearance and low biological activity. Due to their highly desired qualities as drug delivery vehicles, exosomes can be used for the delivery peptides or synthetic drugs for eye disease (101, 102). MSC-exos carrying miRNA-126 could reduce hyperglycemia-induced retinal inflammation by inhibiting the high-mobility group box 1 signaling pathway (103). Exosome-carried adeno-associated virus type 2 showed high efficiency in retinal transduction (104). Therefore, MSC-exos may presumably provide a curative option for SS-associated dry eyes. While, exosomes have shown promising results for potential therapeutic applications, most of these therapeutic effects have only been observed in the experimental stage, and there is a long way to go before reaching large-scale clinical application.

## Conclusion and Perspectives

For decades, researchers have been struggling to develop superior diagnostic and treatment methods for patients with SS. Accumulating evidence has indicated that exosomes may play an important role in the pathophysiology of autoimmune disorders. In this review, we have summarized exosome-mediated effects mediated in SS, the potential of exosomes as biomarkers, as well as their potential therapeutic uses. Nevertheless, gaps remain in the understanding of exosome biogenesis and action. The fundamental mechanisms of exosomes utilized as biomarkers and therapeutic nanocarriers in SS and other autoimmune diseases are not fully understood. In the future, the use of exosomes for SS and other autoimmune diseases will face several challenges that will require further detailed exploration. First, methods for the detection, separation, and purification of exosomes and exosomal miRNA are relatively cumbersome and complicated at present. Thus, there is a need for simplified, cost-effective, and reproducible techniques. Moreover, appropriate production and storage methods for exosomes are critical for preserving their biological activity and are thus essential for obtaining high-quality exosomes. Existing methods are more or less insufficient in obtaining and preserving high yields of purified exosomes. In addition, it is important to establish robust ways to evaluate the effects of exosomal treatment *in vivo*. Despite challenges in the use of exosomes, these vesicles have shown great potential within the biomedical field. As technology advances, the above-mentioned limitations will be resolved, and exosomes may be utilized for novel and advanced therapies. Altogether, both basic and applied research on exosomes in SS is still at an early stage, requiring further investigation.

## Author Contributions

YH, RL, GY, and QX designed and revised the review manuscript, and approved the final manuscript. YH and RL wrote the review manuscript. SY and SL helped in finding references. All authors contributed to the article and approved the submitted version.

## Conflict of Interest

The authors declare that the research was conducted in the absence of any commercial or financial relationships that could be construed as a potential conflict of interest.
